# Monthly Record of Deaths in the City of Buffalo

**Published:** 1857-11

**Authors:** C. L. Dayton

**Affiliations:** Health Physician


ART. IX.— *Report of Mortality in Buffalo for the month of Sept.*, 1857. By C. L. Dayton, M. D., Health Physician.

| DISEASES. | No. | Males. | Females. |
|---|---|---|---|
| Accident, | 2 | 2 | |
| Asphyxia Immersorum, | 6 | 6 | |
| Apoplexia, | 1 | | 1 |
| " Pulmonum, | 1 | | 1 |
| Bronchitis, | 2 | 2 | |
| Coup de Soleil, | 1 | 1 | |
| Cholera Infantum, | 45 | 26 | 15 |
| Carcinoma, | 4 | 2 | 2 |
| Croup, | 3 | 1 | 2 |
| Cancrum Oris, | 1 | | 1 |
| Cephalitis, | 4 | 2 | 2 |
| Dyspepsia, | 1 | I | |
| Diarrhœa, | 26 | 16 | 10 |
| Dysenteria, | 18 | 10 | 8 |
| Dentition, | 14 | 7 | 7 |
| Debilitas Senilis, | 3 | 1 | 2 |
| Enteritis, | 1 | | 1 |
| Eclampsia, | 23 | 10 | 13 |
| " Puerperalis, | 1 | | 1 |
| Epilepsy, | 2 | 2 | |
| Febris Typhoid, | 5 | 2 | 3 |
| " Remittent, | 2 | 1 | 1 |
| " Scarlatina, | 6 | 2 | 3 |
| Fractured Femoris, | 1 | 1 | |
| Gastritis, | 1 | 1 | |
| " et Ulceratio, | 1 | | 1 |

REGISTER OF MORTALITY—CONTINUED.

| DISEASES. | No. | Males. | Females. |
|---|---|---|---|
| Hydrops, | 5 | 5 | |
| Hydrocephalus, | 8 | 4 | 3 |
| Hæmoptisis, | 1 | 1 | |
| Hernia, | 1 | 1 | |
| Intemperance, | 2 | 1 | |
| Ignotus, | 19 | 7 | 11 |
| Marasmus, | 20 | 12 | 8 |
| Murder, | 1 | 1 | |
| Morbus Cordis, | 4 | 3 | |
| " Hepatitis, | 1 | | 1 |
| " Brightii, | 1 | | 1 |
| Paralysis, | 1 | 1 | |
| Premature Birth, | 1 | 1 | |
| Pertussis, | 7 | 3 | 4 |
| Pneumonitis, | 5 | 5 | |
| Purpura, | 1 | 1 | |
| Rheumatism, | 1 | 1 | |
| Rubeola, | 2 | 2 | |
| Spina Bifida, | 1 | 1 | |
| Scrophula, | 1 | 1 | |
| Still-born, | 10 | 6 | 4 |
| Stomatitis Materna, | 1 | | 1 |
| Starvation, | 1 | 1 | |
| Tuberculosis Pulmonalis, | 20 | 12 | 8 |
| Total, | 290 | | |

SEXES.

| | |
|---|---|
| Males, | 165 |
| Females, | 115 |
| Sex not given, | 10 |
| Total, | 290 |

AGES.

| | | | |
|---|---|---|---|
| Still-born, | 9 | Between 20 years and 30 years, | 20 |
| 1 day, | 2 | " 30 " " 40 " | 19 |
| 1 day and 30 days, | 26 | " 40 " " 50 " | 10 |
| Between 1 month and 6 months, | 43 | " 50 " " 60 " | 10 |
| " 6 months and 12 " | 50 | " 60 " " 70 " | 2 |
| " 1 year " 3 years, | 68 | " 70 " " 80 " | 4 |
| " 3 " " 5 " | 9 | " 80 " " 90 " | 0 |
| " 5 " " 10 " | 7 | " 90 " " 100 " | 0 |
| " 10 " " 20 " | 9 | " 100 " | 0 |
| Total number under 10 years, | 214 | Total number over 10 years, | 74 |

Ages not given, 2 Total, 290

NATIVITIES.

| | | | |
|---|---|---|---|
| American, (white,) | 191 | Prussian, | 1 |
| " (colored,) | 3 | Saxon, | 1 |
| German, | 50 | French, | 3 |
| Irish, | 29 | Scotch, | 2 |
| English, | 4 | Poland, | 1 |
| Canadian, | 0 | Country not given, | 5 |

Total, 290

